# A case of langerhans cell histiocytosis of the mandible that spontaneously regressed after biopsy in a child

**DOI:** 10.1002/ccr3.4321

**Published:** 2021-06-24

**Authors:** Kisho Ono, Tatsuo Okui, Yuki Kunisada, Kyoichi Obata, Masanori Masui, Shoji Ryumon, Soichiro Ibaragi, Tomoya Nakamura, Akira Sasaki

**Affiliations:** ^1^ Department of Oral and Maxillofacial Surgery Dentistry and Pharmaceutical Sciences Okayama University Graduate School of Medicine Okayama Japan; ^2^ Department of Oral and Maxillofacial Surgery Faculty of Medicine Shimane University Shimane Japan

**Keywords:** dentistry, general surgery, oncology

## Abstract

In younger patients of LCH, we should consider that the effectiveness of follow‐up without aggressive treatment for SS‐type LCH in the oral and maxillofacial bone. However, there are very rare case in which an SS‐type LCH recurred after showing a healing tendency. Regular follow‐up must be performed even after healing.

## Graphical abstract

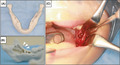

## 1 INTRODUCTION

Langerhans cell histiocytosis (LCH) is a rare tumor, and LCH may develop in the mandible in young people. LCH treatment options include surgery, chemotherapy, and radiation therapy. In some cases, healing may occur spontaneously. We describe a case of LCH in the mandible of a child who experienced spontaneous regression after biopsy.

Langerhans cell histiocytosis (LCH) is a disease in which one type of antigen‐presenting cells, the Langerhans cells, aggregates and proliferates in various organs such as the bone, skin, liver, spleen, or lung, resulting in tissue damage,^1^, ^2^ LCH was previously known as histiocytosis X and was classified into three types: eosinophilic granulomatosis, Hand‐Schüller‐Christian disease, and Letterer‐Siwe disease. These are all characterized by the proliferation of Langerhans cells and have been collectively referred to as LCH.^3^ At present, LCH is classified into three groups: single‐system single‐site (SS type), single‐system multi‐site (SM type), and multi‐system multi‐site (MM type), and treatment differs depending on the location and type of disease.^4^ In Japan, an epidemiological study reported that the SS, SM, and MM types of LCH are diagnosed at almost equal rates.^5^ In patients with the SM or MM type, the typical treatment in Japan is multidrug chemotherapy following the protocol proposed by the Japan LCH Study Group.^6^ However, some SS cases experience spontaneous remission, and no sufficient consensus for treatment has yet been reached,^7^, ^8^ We report a case of LCH in the mandible with spontaneous remission after biopsy.

## 2 CASE REPORT

A 4‐year‐old boy was seen by nearby pediatricians for painful swelling of his left cheek. Computed tomography (CT) and contrast‐enhanced magnetic resonance imaging (MRI) showed osteolysis and mass formation in the left angle of mandible, and he was referred to our hospital with suspected ameloblastoma.

**FIGURE 1:**
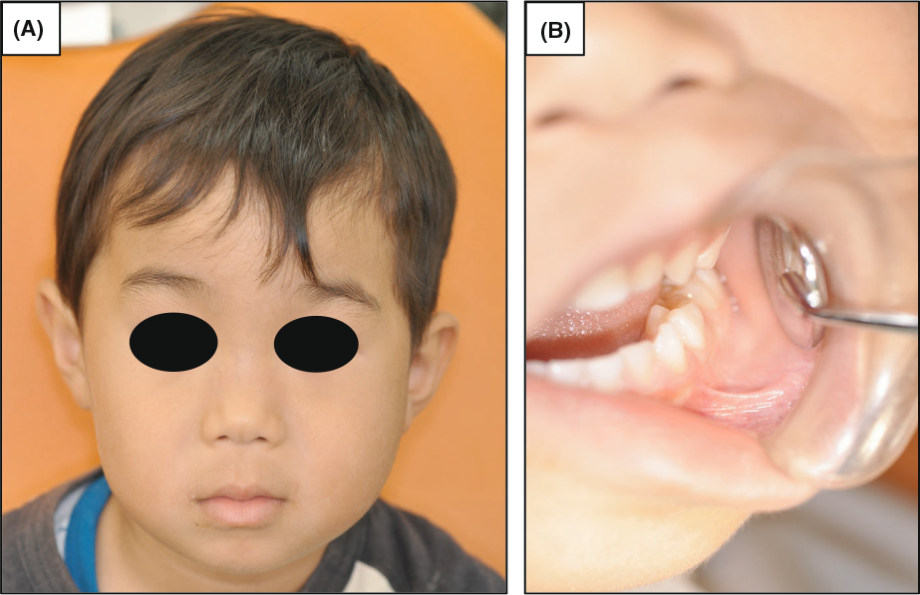
Clinical features at first visit. A, The left mandibular region was diffusely swollen. B, An intraoral photograph shows no obvious abnormality

At the patient's first visit, his left cheek showed painful swelling, and the submandibular lymph nodes were palpable, tender, and mobile (Figure 1). The patient's mouth opening capacity was only about 20 mm, and trismus was recognized.

**FIGURE 2:**
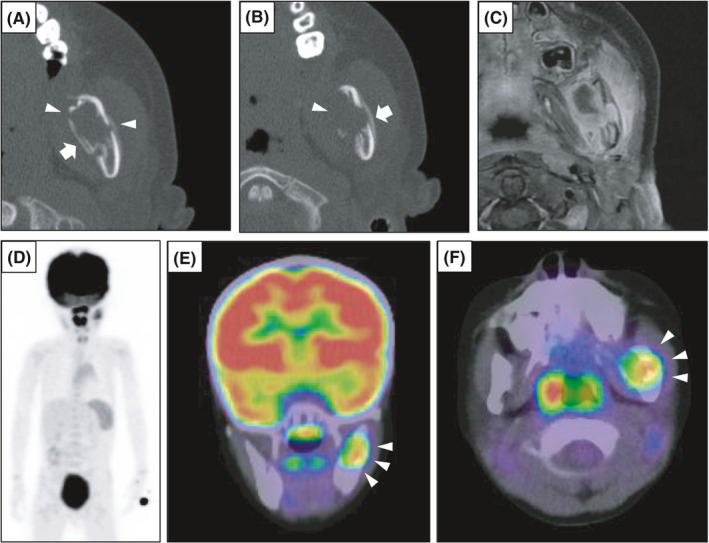
Radiological features. A, B, CT scan showing osteolysis with indistinct borders in the left mandible. A rupture (arrowhead) and periosteal reaction (arrow) was found in the buccolingual cortical bone. C, Contrast‐enhanced MRI T1‐weighted imaging showing a mass extending outside the buccolingual bone. D‐F, Bone scintigraphy and PET‐CT showing abnormal accumulation only in the left mandible (arrowhead)

CT imaging showed osteolysis with indistinct borders in the left mandibular ramus, and rupture was observed in the buccolingual cortical bone (Figure 2A,B). A periosteal reaction was also observed on the buccolingual side. Contrast‐enhanced MRI T1‐weighted images showed that a contrast‐enhancing mass had spread outside the bone on the buccolingual side, and inflammatory edema changed in the surrounding soft tissue (Figure 2C). An unstained area was observed inside the lesion on a T1‐weighted image. Bone scintigraphy showed abnormal accumulation only in the left mandible (Figure 2D). Positron emission tomography (PET)‐CT also showed abnormal accumulation of ^18^F‐fluorodeoxyglucose (FDG) (SUV_max_ = 4.90) only in the left mandible (Figure 2E,F). These imaging findings were atypical for ameloblastoma. The clinical differential diagnosis included LCH, myeloma, lymphoma, and plasmacytoma, but all were atypical and histopathological examination was required.

**FIGURE 3:**
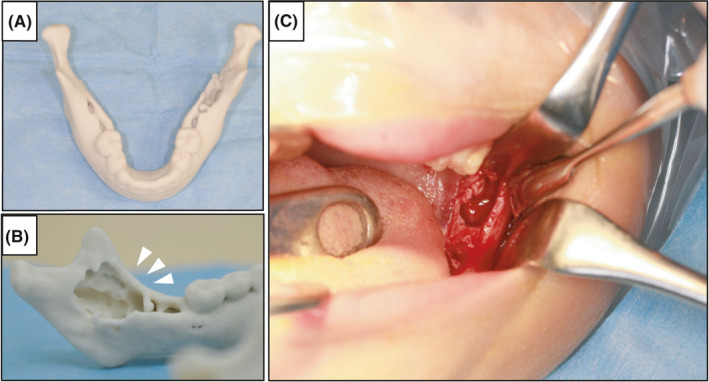
A, B, Three‐dimensional model showing osteolysis in the left mandible. Arrowheads indicate the location of the lesion. C, Intraoperative photograph at biopsy

**FIGURE 4:**
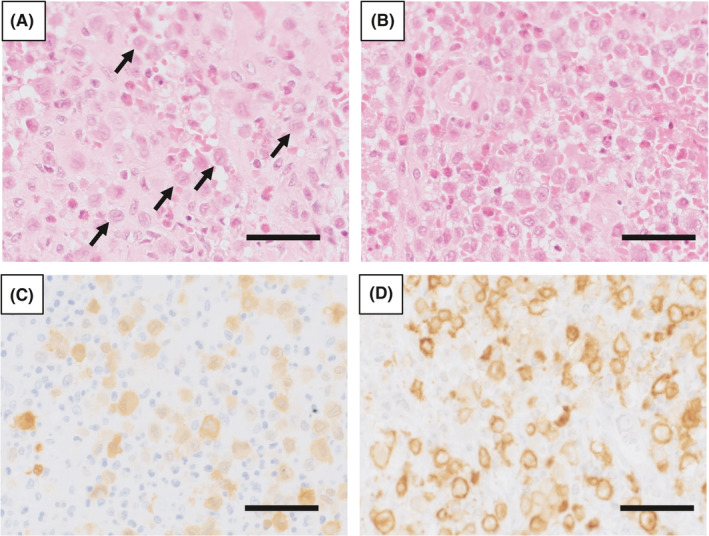
Histologic features. A, B, Hematoxylin and eosin staining showing proliferation of Langerhans cells (arrows) with large and distinct cytoplasm and coffee bean‐like nuclei (A) and infiltration of many eosinophils (B). Scale bar: 50 μm. C, D, Immunohistochemical staining showing immunoreactivity for S‐100 (C) and CD1a (D). Scale bar: 50 μm

To establish a diagnosis, an incisional biopsy was performed under general anesthesia (Figure 3). Specifically, after infiltration anesthesia, a mucosal periosteal flap was created by making an incision in the distal direction from the buccal gingiva of the left mandibular second deciduous molar along the lateral oblique line. When the tumor film located inside the lateral oblique line was peeled off, a light brown exudate was observed from the inside. Tumors taken during the biopsy showed granulation‐like tissue (sample size: 8x8mm). Proliferation of Langerhans cells with large and distinct cytoplasm and coffee bean‐like nuclei was observed by hematoxylin and eosin staining (Figure 4A). Additionally, many eosinophilic infiltrates were involved, and some areas showed bleeding, hyperplasia of the capillaries, congestion, and necrotic tissue (Figure 4B). Immunohistochemical (IHC) staining identified these clusters of cells as Langerhans cells due to their intense immunoreactivity for S‐100 protein and CD1a (Figure 4C,D). Based on these histopathological findings, together with the tumor morphology, developmental site, and markers, we diagnosed this patient with SS‐type LCH.

**FIGURE 5:**
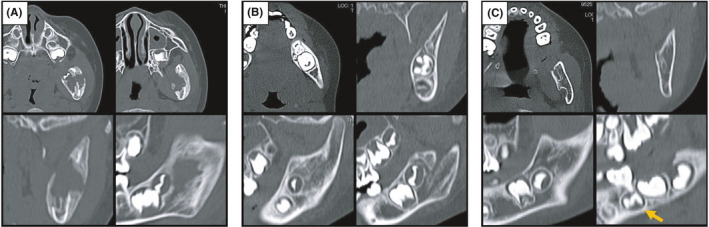
CT images during follow‐up at 2 mo (A), 6 mo (B), and 18 mo (C) after biopsy. Tooth germ formation of the left lower second premolar was found at a site within the original lesion range (yellow arrow)

Surgical intervention was initially planned; however, because the patient was a child, factors such as jaw growth and tooth formation had to be taken into consideration. On the other hand, there have been reports of cases in which spontaneous remission can be expected in SS‐type LCH at the bone limit.^7^ When we performed follow‐up, the swelling and tenderness of the left cheek disappeared with the disappearance of acute inflammation after biopsy. CT scans taken two months after the biopsy showed no apparent exacerbation of the lesions (Figure 5A), and a CT scan at six months showed a marked reduction in the size of the lesion, regeneration of the buccolingual cortex, and no buccolingual periosteal response (Figure 5B). In addition, tooth germ formation of the left lower second premolar was observed in the lingual cortical bone. Consequently, the lesion was considered to be healing, and we followed it without additional treatment. Eighteen months after the biopsy, the lesion had completely disappeared (Figure 5C). Since then, we have continued regular follow‐up, and at the time of writing, it has been 7 years with no sign of relapse or onset in other organs.

## 3 DISCUSSION

LCH is a condition that results from the monoclonal expansion of immunophenotypically and functionally immature Langerhans cells.^9^ In recent years, Langerhans cells present in LCH lesions have come to be referred to as LCH cells and are often distinguished from those in the epidermis because there are obvious differences in phenotype, genetic abnormality, and morphological findings,^10^, ^11^ The cause of LCH is unknown, and it is not still fully understood whether it is neoplastic or responsive. LCH cells tend to have mutations in the BRAF gene. Therefore, it is considered that the therapeutic effect of BRAF‐specific agents can be expected.^12^ It has been reported that vemurafenib monotherapy for high‐risk infant LCH who was positive for BRAF V600E mutation and refractory to vinblastine and cladribine was rapidly effective.^13^ Some research mentioned that LCH may have a neoplastic aspect.^14^ It was reported that the BRAF V600E mutation, which is a carcinogenic mutation, was found in LCH cells in about half of the cases and that phosphorylation of ERK protein was enhanced in almost all cases even in cases without BRAF mutation.^15^ On the other hand, there was reported that LCH may mimic several oro‐facial inflammatory and neoplastic diseases.^16^ At the lesions of LCH, not only LCH cells, but also various inflammatory cells such as eosinophils, lymphocytes, macrophages, and osteoclast‐like polynuclear giant cells are infiltrated. Serum from LCH patients has elevated levels of the soluble interleukin‐2 (IL‐2) receptor, a T‐cell activation marker, and receptor activator of nuclear factor kappa‐B ligand (RANKL), an osteoclast activator, by reflecting cytokines/chemokines secretion from the lesion site.^17^ Especially in high‐risk LCH patients, osteopontin (OPN), IL‐18, and CCL2, which are representative of inflammatory cytokines/chemokines, are elevated.^18^ Excessive secretion of cytokines/chemokines and activation of osteoclasts are expected to lead to tissue destruction.^19^ LCH is classified into three types according to the site of occurrence and disease type. In the present case, SS‐type LCH, known as eosinophilic granulomatosis, developed independently in the mandible. The frequencies of occurrence of the SS, SM, and MM types are almost identical, and as the types become more multiple and/or multi‐organ, the patients tend to be younger, and recurrence and mortality tend to increase.^4^ LCH can occur in various sites, and 78% of LCH patients have bone lesions.^4^ Among the incidence of bone lesions, it has been reported that the mandible accounts for about 7% to 9% of cases and the maxilla accounts for about 1%.^4^

The clinical manifestations of LCH in the jawbone include bone pain and swelling, trismus, tooth sway, pathological fractures, and bone deformities.^20^ Imaging examinations show a solitary or multiple bone punching or bone destruction with a periosteal reaction,^21^, ^22^ However, it is not easy to discriminate this disease from clinical and imaging findings, and the definitive diagnosis is based on pathological findings.^23^ LCH histopathology shows diffuse or focal growth of LCH cells with characteristic coffee bean‐like nuclei and eosinophilic cytoplasm, accompanied by eosinophil infiltration. LCH is immunohistologically positive for S‐100 protein and CD1a and is characterized by the presence of Birbeck granules in the cytoplasm when observed with an electron microscope.^24^

The choice of treatment for LCH depends on the type of disease. For the SM and MM types, combination chemotherapy is often selected. However, the disease can progress rapidly and lead to a fatal progress in refractory and recurrent cases, and the definitive establishment of a treatment method for such cases is much desired. The treatment of SS‐type bone lesions, on the other hand, has been performed empirically and has included surgical curettage, local administration of steroids, combination chemotherapy, and radiation therapy; there is no consensus on treatment to date. It has been reported that a quarter of SS‐type bone lesions are expected to regress spontaneously.^25^, ^26^ However, chemotherapy is often selected when there are craniofacial lesions other than the canopy, which are considered to be at high risk of developing diabetes insipidus.^5^ Besides, it has been suggested that lesions smaller than 2 cm should be completely curetted at biopsy, lesions measuring 2‐5 cm should be partially curetted at biopsy, and lesions larger than 5 cm should be observed at biopsy to track their progress.^25^ There have been few reports of spontaneous regression after biopsy of SS‐type LCH in the mandible,^7^, ^27^, ^28^, ^29^ In the present case, although the lesion showed marked progression, we decided to perform follow‐up, considering the patient's age and the extent of the lesion. At 6 months after the biopsy, spontaneous regression of the lesion was observed, and no recurrence or complications have been observed up to the present time, 7 years after the biopsy.

Various hypotheses have been proposed for spontaneous regression of LCH, including arrested lesion growth due to the disappearance of local inflammation,^30^ decompression of lesions by biopsy,^27^ triggered apoptosis of tumor cells,^31^ and more. Although we were unable to clarify the reason for spontaneous regression in the present case, our experience suggests the effectiveness of follow‐up without aggressive treatment for SS‐type LCH in the jawbone. However, there has been a report of a very rare case in which an SS‐type LCH recurred after showing a healing tendency and progressed rapidly to a fatal outcome.^32^ Furthermore, even with SS‐type LCH, cases in craniofacial bone with soft tissue masses convey higher risk of future diabetes insipidus and central nervous system disorders compared with cases with lesions in other bones,^33^, ^34^ It is recommended that regular follow‐up be performed even after healing.

## CONFLICTS OF INTEREST

The authors declare that they have no conflicts of interest regarding the publication of this paper.

## AUTHOR CONTRIBUTIONS

KO, TO, SI and TN: gathered the patient data, performed a literature review, and wrote the manuscript. YK, KO, MM and SR: reviewed, corrected patient data, and revised the manuscript. AS: was involved in overall supervision of the paper. All authors: read and approved the final manuscript.

## CONSENT STATEMENT

Written consent was obtained from the patient's parents.

## ETHICAL APPROVAL

This case report did not receive any funding. Authors have access to all source data for this case report. All procedures performed in this study were in accordance with the ethical standards of Okayama University Research Ethics Committee and with the 1964 Helsinki Declaration and its later amendments.

## Data Availability

Further supporting data are available from the authors on request.
